# Features of internal absorbed dose microdistribution in biological tissue irradiated by ^31^SiO_2_ microparticles compared with dose microdistribution from exposure to ^56^MnO_2_ particles

**DOI:** 10.1093/jrr/rrae096

**Published:** 2024-12-19

**Authors:** Valeriy Stepanenko, Andrey Kaprin, Sergey Ivanov, Peter Shegay, Viktoria Bogacheva, Sergey Shinkarev, Hitoshi Sato, Noriyuki Kawano, Megu Ohtaki, Nariaki Fujimoto, Satoru Endo, Aya Sakaguchi, Evgenia Ostroumova, Kassym Zhumadilov, Almagul Kushugulova, Masaharu Hoshi

**Affiliations:** A. Tsyb Medical Radiological Research Center - Branch of the National Medical Research Radiological Centre of the Ministry of Health of the Russian Federation, 4 Koroleva St., Obninsk, Kaluga Region 249036, Russian Federation; National Medical Research Radiological Centre of the Ministry of Health of the Russian Federation, Koroleva Str., 4., Obninsk, Kaluga Region 249036, Russian Federation; Peoples’ Friendship University of Russia, 6 Miklukho-Maklaya St., Moscow 117198, Russian Federation; P.A. Hertzen Moscow Oncology Research Institute-branch of the National Medical Research Radiological Centre of the Ministry of Health of the Russian Federation, 2nd Botkinsky Drive 3, Moscow 125284, Russian Federation; A. Tsyb Medical Radiological Research Center - Branch of the National Medical Research Radiological Centre of the Ministry of Health of the Russian Federation, 4 Koroleva St., Obninsk, Kaluga Region 249036, Russian Federation; Peoples’ Friendship University of Russia, 6 Miklukho-Maklaya St., Moscow 117198, Russian Federation; National Medical Research Radiological Centre of the Ministry of Health of the Russian Federation, Koroleva Str., 4., Obninsk, Kaluga Region 249036, Russian Federation; A. Tsyb Medical Radiological Research Center - Branch of the National Medical Research Radiological Centre of the Ministry of Health of the Russian Federation, 4 Koroleva St., Obninsk, Kaluga Region 249036, Russian Federation; State Research Center - Burnasyan Federal Medical Biophysical Center of Federal Medical Biological Agency, 46 Zhivopisnaya St., Moscow 123098, Russian Federation; Ibaraki Prefectural University of Health Sciences, 4669-2 Ami, Ami-machi, Inashiki-gun, Ibaraki 300-0394, Japan; The Center for Peace, Hiroshima University, Higashisenda-machi 1-1-89, Naka-ku, Hiroshima 730-0053, Japan; The Center for Peace, Hiroshima University, Higashisenda-machi 1-1-89, Naka-ku, Hiroshima 730-0053, Japan; Research Institute for Radiation Biology and Medicine, Hiroshima University, 1-2-3, Kasumi, Minami-ku, Hiroshima 734-8551, Japan; Graduate School of Advanced Science and Engineering, Hiroshima University, 1-4-1, Kagamiyama, Higashi, Hiroshima 739-8527, Japan; Institute of Pure and Applied Sciences, University of Tsukuba 1-1-1 Tennodai, Tsukuba, Ibaraki 305-8577, Japan; Environment and Lifestyle Epidemiology Branch, International Agency for Research on Cancer/WHO, 25 avenue Tony Garnier, Lyon 69366, France; International Department of Nuclear Physics, New Materials and Technology, L. N. Gumilyov Eurasian National University, Munaitpasova 13, office 300, Astana, Kazakhstan; Nazarbayev University, 53 Kabanbay Batyr Avenue, Nur-Sultan city 010000, Kazakhstan; The Center for Peace, Hiroshima University, Higashisenda-machi 1-1-89, Naka-ku, Hiroshima 730-0053, Japan

**Keywords:** internal irradiation, ^31^Si, ^56^Mn, radioactive microparticles, lungs, alveoli, bronchioles, radiation dose microdistribution

## Abstract

Radiobiological studies are ongoing to understand the consequences of internal exposure to neutron-activated radioactive microparticles, which were sprayed over experimental rats and mice. Special attention in these experiments is given to internal irradiation with radioactive microparticles with short-lived neutron-activated radionuclides ^31^Si (T_1/2_ = 2.62 h) and ^56^Mn (T_1/2_ = 2.58 h), which are among the main dose-forming factors from residual radioactivity activated in soils by neutrons in the first hours after atmospheric nuclear explosions. The presented work is devoted to microdosimetry peculiarities of ^31^SiO_2_ and ^56^MnO_2_ microparticles. The radiation from ^31^Si consists of intensive short-range beta particles and gamma rays with very low intensity. It differs from the radiation of ^56^Mn, which includes intensive beta particles, low energy Auger electrons and very intensive gamma rays. Differences in the energies and intensities of short-range beta particles and penetrating gamma rays emitted by ^31^SiO_2_ and ^56^MnO_2_ microparticles can lead to differences in the spatial microdistribution of absorbed dose around the corresponding radioactive microparticles embedded in biological tissue. It was found in the presented work that the absorbed doses of beta radiation emitted by ^56^MnO_2_ and ^31^SiO_2_ microparticles has significant but different spatial gradients with distances in biological tissue that correspond to the typical thickness of epithelial cells of lungs’ alveoli and bronchioles. The results obtained are necessary for a better understanding of radiobiological effects of internal exposure by radioactive microparticles with ^56^Mn and ^31^Si observed in framework of performed and ongoing radiobiological studies with experimental animals—rats and mice.

## INTRODUCTION

Radiobiological studies with experimental animals were launched to understand the consequences of internal exposure by neutron-activated radioactive microparticles [1, 2]. Special attention was paid to internal irradiation by radioactive microparticles with short-lived neutron-activated radionuclides ^31^Si (T_1/2_ = 2.62 h) and ^56^Mn (T_1/2_ = 2.58 h), which were sprayed over experimental rats and mice [2–15]. These radionuclides belong to the main dose-forming factors from residual radioactivity activated in soils by neutrons during the first hours after the atomic explosions [16–21]. One of the important reasons for the interest in studying the radiobiological effects and features of absorbed dose forming at internal irradiation by short-lived neutron-activated radionuclides is the published information on the observed health effects among persons who arrived to Hiroshima on the first day after the atomic bombing on August 6, 1945 [22–25]. These people were not exposed to direct gamma-neutron radiation at the moment of atomic explosion, but, nevertheless, they showed syndromes similar to the consequences of radiation exposure, which was presumably due to residual radioactivity [22, 24, 25]. Interest in these effects is in the focus of scientific discussions [1, 2, 20–25], because the impact of residual radiation (especially products of neutron activation) shortly after atomic explosion was not well assessed [26–30]. Radiation emission of ^31^Si consists of intensive short-range beta-particles (mean energy 0.595 MeV with 100% intensity) and gamma-rays with very low intensity (0.07% with energy of 1.27 MeV) [31, 32]. It is differ from radiation emission of ^56^Mn, which includes intensive beta-particles (mean energy 0.829 MeV, 100% intensity) and more intensive gamma-rays (143% intensity, mean energy 1.19 MeV) [31, 32]. The difference in energies and intensities of short-range beta-particles and penetrating gamma-rays emitted by ^31^Si and ^56^Mn can lead to the difference in spatial microdistribution of internal absorbed dose around radioactive microparticles in cases when SiO_2_ or MnO_2_ particles are incorporated into the body in the experiments with animals’ internal irradiation by sprayed radioactive microparticles containing neutron-activated ^31^Si or ^56^Mn. This can be the reason of differences in radiobiological effects investigated in the corresponded experiments with animals. Here we reported on peculiarities of spatial dose microdistribution around ^31^SiO_2_ microparticles incorporated into biological tissue compared to dose microdistribution around ^56^MnO_2_ particles. The study findings can be useful for better understanding of effects observed in performed and ongoing radiobiological experiments with animals (rats and mice) internally irradiated by different neutron-activated radioactive microparticles (^31^SiO_2_ and ^56^MnO_2_) [1, 2].

## MATERIALS AND METHODS

Radial absorbed dose distributions in the biological tissue around ^31^SiO_2_ and ^56^MnO_2_ microparticles, were calculated using Monte-Carlo code MCNP-4C [33] with MCPLIBO2 library. All types, energies and intensities of ^31^Si and ^56^Mn ionizing radiation were accounted for in these dose calculations. Components of ^31^Si beta-particles and photon radiation are presented in Tables 1 and 2 [31, 32]. Information regarding ^56^Mn ionizing radiation emission is presented in Tables 3–6 [31, 32]. For dose calculations continuous spectra of ^31^Si and ^56^Mn beta-particles [32] were approximated by discrete spectra of monoenergetic electrons. It should be noted that for electron energies less than 10 keV, the dose calculations were performed using information about dose point kernels for low-energy electrons presented in Berger [34].

**Table 1:** Beta-particles emission from ^31^Si decay [31, 32]

Mean / Max energy (MeV)	Intensity, beta-particle per decay
0.0687 / 0.22588	0.0007
0.5956 / 1.49203	0.9993

**Table 2:** Gamma- and X-ray radiation from ^31^Si decay [31, 32]

Emission type	Emission energy (MeV)	Intensity, photon per decay
X-ray	0.002010	2.50 × 10^−10^
	0.002136	5.33 × 10^−10^
gamma	1.26612	0.0007

**Table 3:** Beta-particles emission from ^56^Mn decay to excited levels of ^56^Fe [31, 32]

Mean / Max energy (MeV)	Intensity, beta-particle per decay
0.0736 / 0.2502	0.0002
0.0992 / 0.3257	0.0116
0.1905 / 0.5726	0.0004
0.2553 / 0.7356	0.1460
0.3820 / 1.0379	0.2790
0.6364 / 1.6104	0.0006
1.2170 / 2.8487	0.5630

**Fig. 1:**
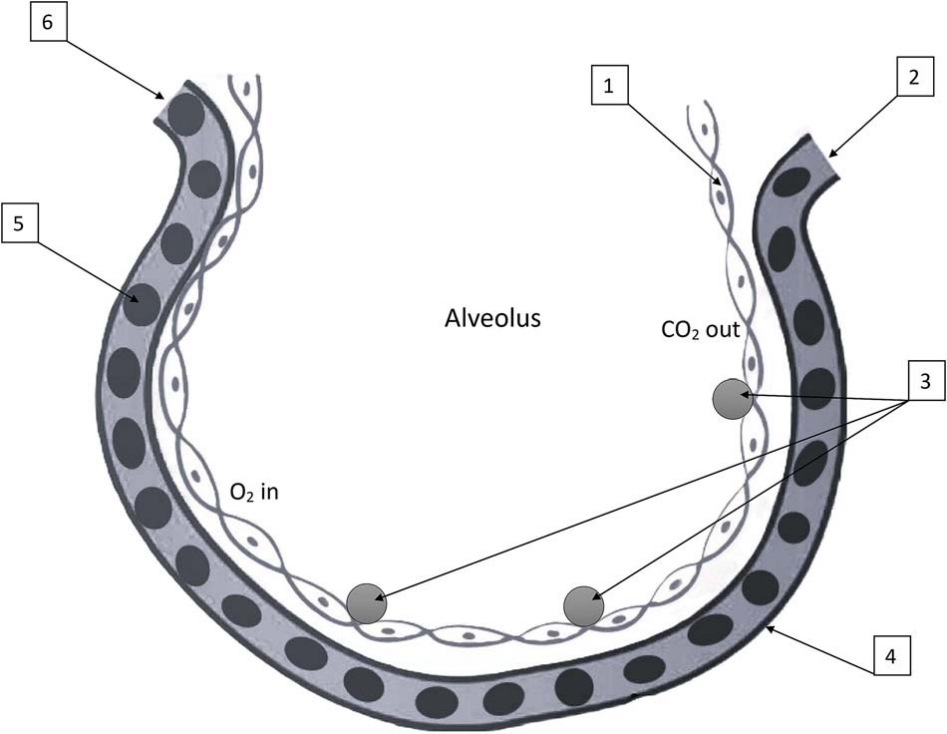
Schematic representation of lung’s alveolus [39] with radioactive microparticles attached to the layer of simple squamous epithelium cells. 1 – layer of simple squamous epithelium cells 0.2 μm thick covered by layer of surfactant 0.01 μm thick; 2 – pulmonary capillary arterial end; 3 – radioactive microparticles (^31^SiO_2_ or ^56^MnO_2_) attached to layer of epithelium cells; 4 – alveolar capillary membrane; 5 – red blood cells; 6 – pulmonary capillary venous end.

**Fig. 2:**
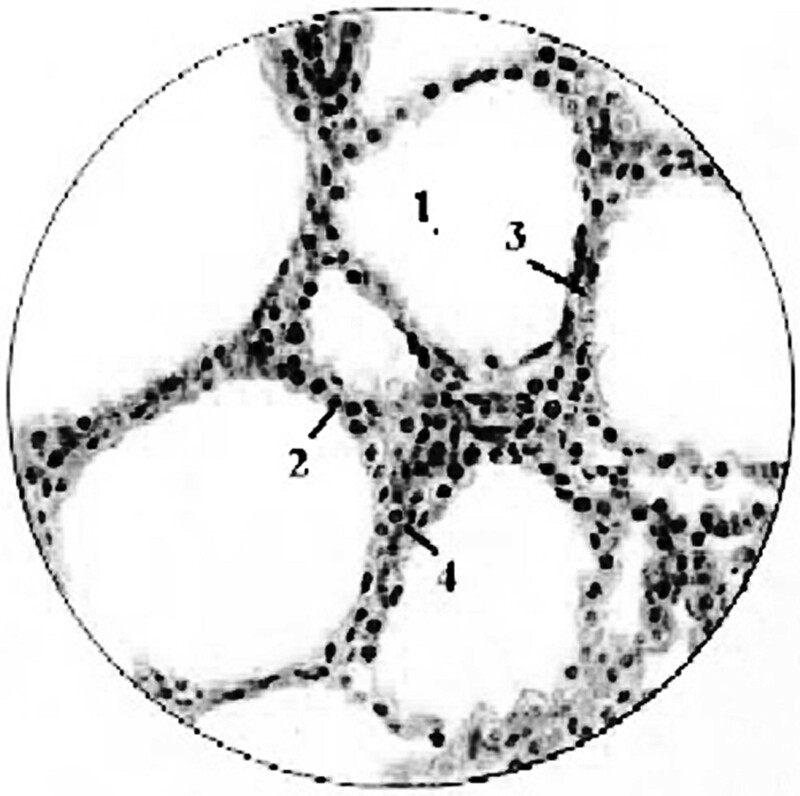
Alveoli with simple squamous epithelia cells (histological image). 1- alveoli, 2- cells lining the alveoli are the parts of thin single-layer squamous epithelium, 3- interalveolar septa, 4- blood capillaries.

Similar to Stepanenko *et al.* [8, 35], the geometry in which the transport of electrons and photons was simulated is the follows: there are spherical layers of homogeneous biological tissue around ^31^SiO_2_ and ^56^MnO_2_ microparticles. The radioactive microparticles are located in the center of the surrounding spherical layers of biological tissue. These radioactive microparticles assumed to be as a uniform spherical radioactive sources with diameters of 2.4 μm for SiO_2_ (Admatechs, Japan), and 3 μm for MnO_2_ (Rare Metallic Co., Ltd., Japan). The densities of SiO_2_ and MnO_2_ are equal to 2.2 g/cm^3^ and 5.03 g/cm^3^, respectively. The activities of neutron activated ^31^Si and ^56^Mn radionuclides were assumed to be uniformly distributed in the volumes of SiO_2_ and MnO_2_ microparticles. Absorption of beta radiation inside the volumes of SiO_2_ and MnO_2_ particles was accounted for. In such spherical and uniform geometries only one parameter is important for the calculation of absorbed dose distribution in biological tissue around ^31^SiO_2_ and ^56^MnO_2_ microparticles—it is the radial distance from the surface of radioactive particle to the sites of irradiation [4, 8, 35]. The absorbed dose around ^31^SiO_2_ and ^56^MnO_2_ microparticles was calculated in spherical layers of homogeneous biological tissue at radial distances from the surface of microparticles ranged from 10^−2^ μm to 10^4^ μm (10^4^ μm = 1 cm). Similar to Stepanenko *et al.* and Ohtaki *et al.* [4, 8], the accounting for microstructure of lung tissues was not needed in this approach. The density and composition of biological tissue was taken from ICRP Publication 89 [36]. It was accepted that as a result of inhalation, ^31^Si dioxide and ^56^Mn dioxide microparticles were directly deposited to the epithelium of lung’s alveoli and bronchioles (see Fig. 1). Epithelium cells are considering as the closest and most irradiated ‘targets’ of radiation exposure from radioactive particles deposited in alveoli and bronchioles. Similar to Stepanenko *et al.* and Ohtaki *et al.* [8, 23], the following suggestion was accepted for dose calculations: epithelium cells are parts of the layers of biological tissue located just near the radioactive microparticles. According to Katsumiti *et al.* and Maynard and Downes [37, 38], the following thicknesses of epithelium cells were accepted: simple squamous epithelium cells of alveoli—their thickness is not more than 0.2 μm (covered by layer of surfactant about 0.01 μm thick); epithelium cells of bronchioles—thickness is about 10 μm. Figure 1 shows, as an example, a schematic representation of lung’s alveolus with radioactive microparticles deposited on the layer of simple squamous epithelium cells. Figure 2 shows the typical histological image of alveoli with simple squamous epithelia, interalveolar septa and blood capillaries [39].

## RESULTS

**Fig. 3:**
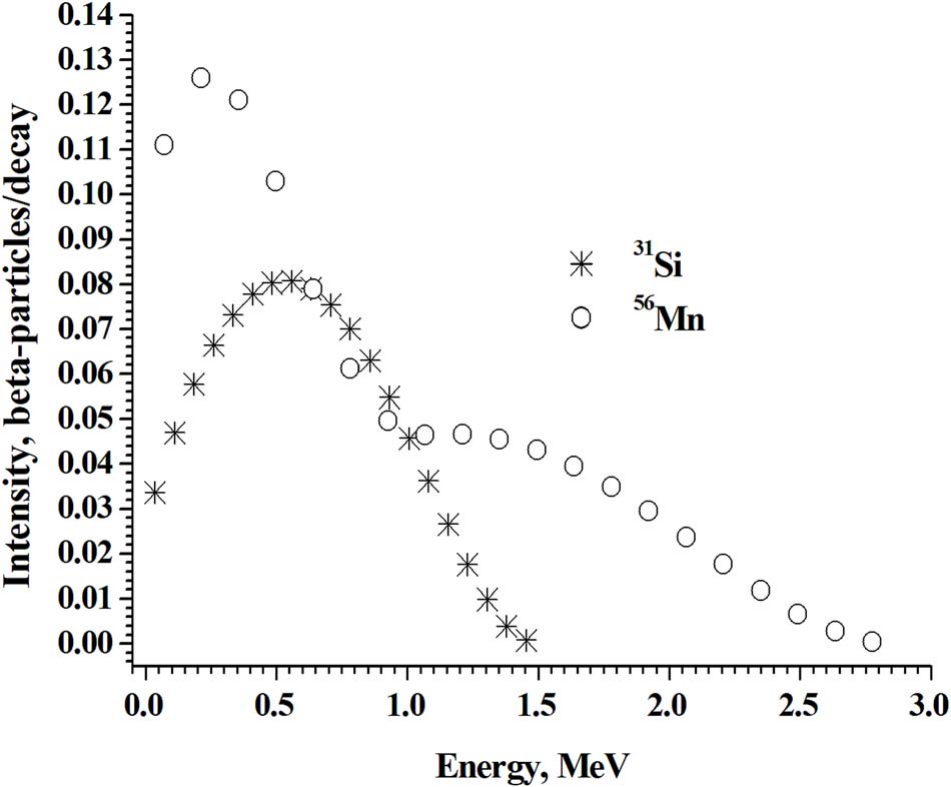
Comparison between discrete spectra of electrons, which represent the continuous beta-particles spectra of ^31^Si and ^56^Mn (indicated by symbols of stars and circles, respectively).

Figure 3 shows discrete spectra of electrons, which represent the continuous beta-particles spectra of ^31^Si and ^56^Mn.

**Fig. 4:**
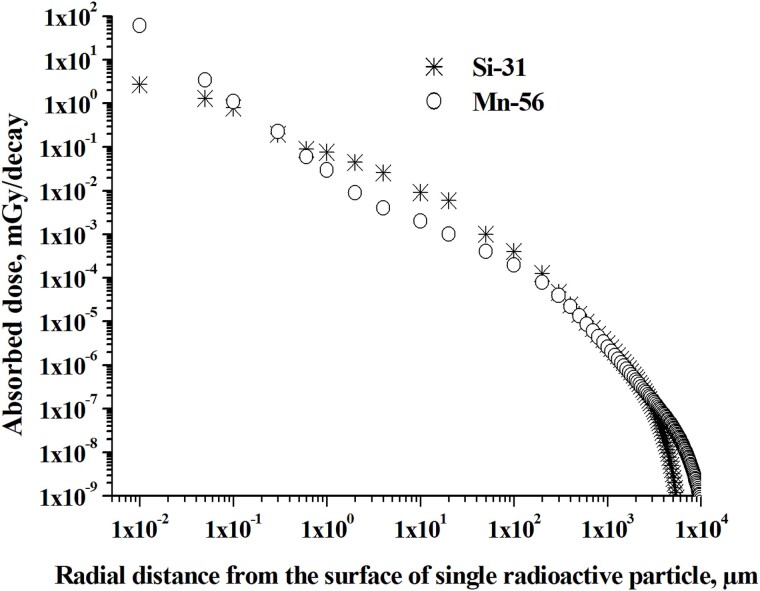
Absorbed dose distribution with distance around single ^31^Si microparticle in comparison with ^56^Mn microparticle, indicated by symbols of stars and circles, respectively (beta-particles).

The low-energy part of the energy spectrum of ^56^Mn beta-particles is significantly more intense when compared with the spectrum of ^31^Si beta-particles (Fig. 3). It is because of multiple ^56^Mn beta emission spectra while ^31^Si has only a single high energy beta spectrum. This may be the reason that directly near the ^56^Mn microparticles the absorbed dose from low-energy betas will be significantly higher than near the ^31^Si microparticles. This assumption is confirmed by the results of calculations of spatial distribution of absorbed dose around microparticles (Fig. 4).

Dose calculation was performed in the following radial layers of biological tissue around radioactive microparticle: from 10^−2^ μm to 10 μm of radial distance from microparticle with 0.01 μm steps and from 10 μm to 10^4^ μm radial distance with 1 μm steps. Absorbed dose per single decay of ^56^Mn and ^31^Si from beta-radiation and electrons emitted by ^56^Mn and ^31^Si microparticles has significant gradients with distances, which correspond to typical dimensions of epithelium cells of lung’s alveoli and bronchioles (Fig. 4). Absorbed dose (expressed as mGy per single decay) caused by low-energy parts of beta-radiation spectrum of ^31^Si on the radial distance less than 1 μm from the ^31^SiO_2_ microparticle embedded in biological tissue, is lower than around ^56^MnO_2_ microparticle. On the other hand, starting from the radial distance of 1 μm until 100 μm, absorbed dose from ^31^SiO_2_ microparticle is higher compared with the dose from ^56^MnO_2_ microparticle. The curves in Fig. 4 show the dependence of absorbed doses per single decay of ^56^Mn and ^31^Si from beta-radiation and electrons emitted by ^56^Mn and ^31^Si microparticles at various radial distances from radioactive microparticles. Using the numerical data we obtained (Fig. 4), the values ​​of absorbed doses averaged over the thickness of the epithelial cells of the alveoli and bronchioles were estimated. As a results, it was estimated, that mean dose over maximal thickness 0.2 μm of alveoli epithelium’s cells is equal to 1.3 mGy/decay for ^31^SiO_2_ microparticle, which is much less in comparison with 16 mGy/decay mean dose for ^56^MnO_2_ microparticle. It should be noted that according to DukeMedicine and MedicalPlanet [40, 41], the minimal thickness of simple squamous cells of lungs’ alveoli epithelium is 0.05 μm. The calculated value of absorbed dose for minimal thickness of lungs’ alveoli epithelium is 2.1 mGy/decay for ^31^SiO_2_ microparticle, which is, as in a case of maximal thickness of alveoli epithelium cells, much lower compared with local dose around ^56^MnO_2_ microparticle equal to 33 mGy/decay. As for the epithelium cells of bronchioles with thickness of about 10 μm: mean dose over thickness 10 μm of bronchioles’ epithelium cells is ~9.0 × 10^−3^ mGy/decay for single ^31^SiO_2_ microparticle, compared with the dose of ~2.0 × 10^−3^ mGy/decay from ^56^MnO_2_ microparticle irradiation, which is about 4–5 times higher than the dose from ^56^MnO_2_ particle.

**Fig. 5:**
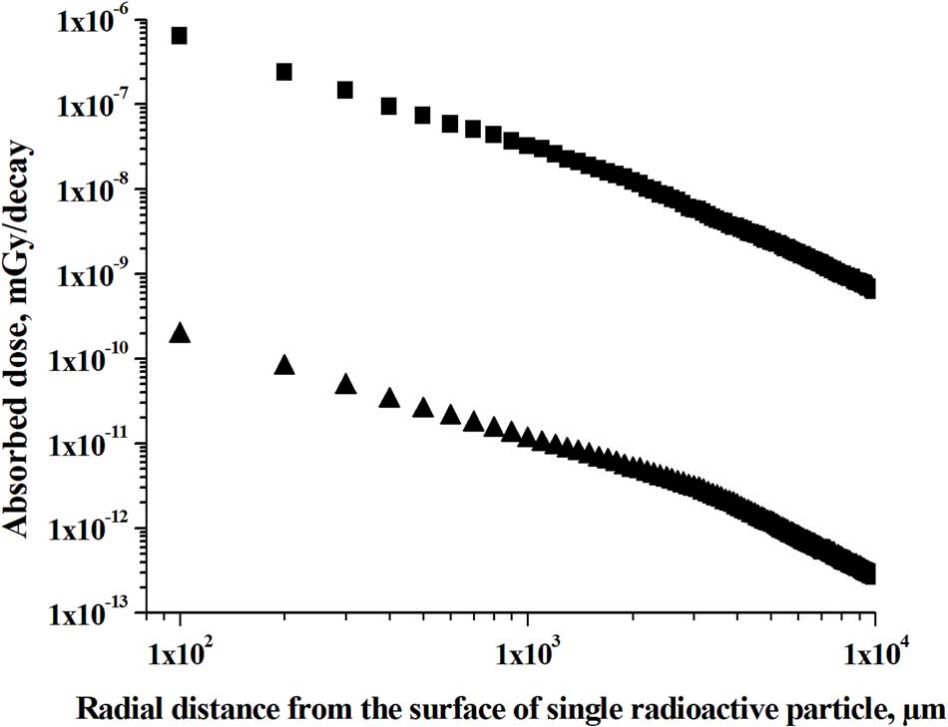
Absorbed dose distributions with distance around single ^56^MnO_2_ and single ^31^SiO_2_ microparticles, indicated by symbols of cubes and triangles, respectively (photon radiation only).

In addition to dose microdistributions from beta-radiation, it is interesting to compare dose microdistributions from photon radiation of single decay of ^31^SiO_2_ and ^56^MnO_2_ radioactive microparticles. Figure 5 shows the results of calculations of the spatial distribution of absorbed dose from photon radiation of single ^31^SiO_2_ and ^56^MnO_2_ microparticles.

Absorbed dose of photon radiation around ^31^SiO_2_ and ^56^MnO_2_ microparticles has significant but different spatial gradients with distances. Analysis of numerical data, expressed in the form of curves presented in Fig. 5, shows that absorbed dose of photon radiation around single ^31^SiO_2_ is negligible compared with ^56^MnO_2_ particle: at a distance of 10^2^ μm, the ratio of the absorbed dose of photon radiation from ^31^Si to that from photon radiation of ^56^Mn is equal to 0.0005 and decreases to 0.0003 at a distance of 10^4^ μm. So, photon radiation doses around single ^31^SiO_2_ microparticle are negligible compared with ^56^MnO_2_ particle, which is due to the very low intensity of photons emitted by ^31^Si.

It may be useful to compare the estimated value of local absorbed doses around single radioactive microparticle at its assumed location near the layer of epithelial cells with the average dose over the volume of the entire organ containing radioactive microparticles distributed throughout the volume of the organ (in our case, these are the lungs of experimental animals).

Indeed, from a general point of view, penetrating gamma radiation can have a significant impact on the average dose on the volume of the entire organ. But it is not comparable to ^31^Si radiation: as shown in Tables 1 and 2, the gamma radiation from ^31^Si is negligible in intensity (0.07%) compared to the beta radiation from ^31^Si (100%). Therefore, this radionuclide is usually classified as a pure beta emitter, and the expected gamma irradiation of the entire organ volume should be significantly less compared to estimates of entire organ doses from ^31^Si beta particles.

As for ^56^Mn, the composition of its radiation is completely different (Tables 3 and 4). The radionuclide ^56^Mn includes intensive penetrating gamma radiation (143% total intensity, average energy 1.19 MeV) and intensive beta radiation (100% intensity, average energy 0.829 MeV). The internal absorbed dose averaged over volume of rats’ lungs irradiated by ^56^MnO_2_ microparticles was estimated in Stepanenko *et al.* [13]. This estimation was performed for ^56^MnO_2_ microparticles suggested to be homogeneously distributed over volume of lungs, and with accounting for total impact from both components of ^56^Mn radiation – beta-particles and gammas. Recalculation of dose presented in units Gy/(kBq × h) [13], to units in mGy/decay, gives the value of 2.33 × 10^−7^ mGy/decay. This is total dose from betas and gammas of ^56^Mn. To estimate dose value, which is related to gamma-radiation only, the ratio of ‘specific absorbed fractions’ of energy from gamma-radiation to beta-radiation of ^56^Mn was evaluated using corresponding information for rat’s lungs (available in Stepanenko *et al.* [32]). This ratio was estimated equal to 0.0105. As the result, internal absorbed dose averaged over volume of rats’ lungs irradiated by only gammas of ^56^MnO_2_ microparticles’s was evaluated to be equal 2.5 × 10^−9^ mGy/decay. This is much less in comparison with 2.0 × 10^−3^ mGy/decay and 9.0 × 10^−3^ mGy/decay, which are local absorbed doses from low-energy parts of beta-radiation spectrum around single ^56^MnO_2_ and single ^31^SiO_2_ microparticles, respectively, in a case of assumed particle’s location on the surface of epithelium cells of bronchioles.

**Table 4:** Gamma radiation from excited levels of ^56^Fe following ^56^Mn beta-decay [31, 32]

Energy of gamma-rays (MeV)	Intensity, photon per decay
0.8468	0.9890
1.0380	0.0004
1.2380	0.0010
1.8110	0.2720
2.1130	0.1430
2.5230	0.0099
2.5980	0.0002
2.6570	0.0065
2.9600	0.0031
3.3700	0.0017

**Table 5:** X-ray radiation from excited levels of ^56^Fe following ^56^Mn beta-decay [31, 32]

Energy of X-ray (MeV)	Intensity, photon per decay
0.00404	5.78 × 10^−5^
0.00639	2.95 × 10^−5^
0.00706	1.19 × 10^−5^

**Table 6:** Auger electrons emission from excited levels of ^56^Fe following ^56^Mn beta-decay [31, 32]

Electrons	Energy, keV	Intensity, electron per decay
L Auger electrons	0.510–0.594	0.000428
K Auger electrons		
KLL	5.370–5.645	0.000139
KLX	6.158–6.400	0.0000382
KXY	6.926–7.105	0.00000261

This means that features of internal absorbed dose microdistribution in biological tissue irradiated by radioactive microparticles may be useful for a better understanding of radiobiological effects of internal exposure by radioactive microparticles with ^56^Mn and ^31^Si.

## CONCLUSIONS AND DISCUSSION

–Absorbed dose from beta-radiation emitted by single ^56^MnO_2_ and ^31^SiO_2_ microparticles has a significant spatial gradient with the distances, which correspond to typical thicknesses of the epithelium cells of lung’s alveoli and bronchioles.

–The main input to the absorbed dose at the level of considered lungs’ microstructures is from the low-energy parts of ^56^Mn and ^31^Si beta-particles spectra.

–It is important to emphasize that local absorbed dose (expressed as mGy per decay) from low-energy parts of beta-radiation spectrum around single ^31^SiO_2_ microparticle in case of assumed particle’s location on the surface of simple squamous cells of lungs’ alveoli epithelium is much lower compared with local dose around ^56^MnO_2_ microparticle: 1.3 mGy/decay for ^31^SiO_2_  *vs* 16 mGy/decay for ^56^MnO_2_ (for maximal thickness of epithelium cells 0.2 μm).

–The calculated value of absorbed dose for minimal thickness of lungs’ alveoli epithelium (0.05 μm) is 2.1 mGy/decay for ^31^SiO_2_ microparticle, which is, as in a case of maximal thickness, much lower compared with local dose around ^56^MnO_2_ microparticle equal to 33 mGy/decay.

–Assuming microparticle’s location on the epithelium cells of bronchioles with thickness of 10 μm, the local radiation dose in epithelium cells of bronchioles from single ^31^SiO_2_ particle is about 4–5 times higher than local dose from ^56^MnO_2_ particle: ~9.0 × 10^−3^ mGy/decay for ^31^SiO_2_ microparticle *vs* ~ 2.0 × 10^−3^ mGy/decay for ^56^MnO_2_.

–Absorbed dose from penetrating photon irradiation in the proximity to the single radioactive microparticles (at the level of considered biological microstructures’ sizes), is much lower (several orders of magnitude) compared to irradiation by beta-particles. Photon radiation doses around single ^31^SiO_2_ microparticles are negligible compared with photon radiation from ^56^MnO_2_ particles, which is due to a very low intensity of photons emitted by ^31^Si.

–Contribution to the absorbed dose from Auger electrons of ^56^Mn at very small distances from the microparticles is negligible because of very low intensity of Auger electrons emission (several orders less in comparison with intensity of beta-particles emission [31, 32, 42]). It should be noted that published data about elevated RBE for Auger electrons and tritiated thimidine [43] are not related to results reported in our paper, as far as high-LET-type cell survival curves have been observed only if Auger-emitters, or low energy beta-emitter tritium were located inside of cell's nucleus (attached to DNA).

The study results are important for comparative analysis and better insight into effects of internal exposure by radioactive microparticles with ^56^Mn and ^31^Si observed in framework of performed and ongoing studies with experimental animals (rats and mice) [1, 2].

## Data Availability

All authors are ready to provide data upon request.
